# Characterization and Kinetic Study of Agricultural Biomass Orange Peel Waste Combustion Using TGA Data

**DOI:** 10.3390/polym17081113

**Published:** 2025-04-19

**Authors:** Suleiman Mousa, Ibrahim Dubdub, Majdi Ameen Alfaiad, Mohammad Yousef Younes, Mohamed Anwar Ismail

**Affiliations:** 1 Chemical Engineering Department, King Faisal University, P.O. Box 380, Al-Ahsa 31982, Saudi Arabia; saamousa@kfu.edu.sa (S.M.); malfaiad@kfu.edu.sa (M.A.A.); myounes@kfu.edu.sa (M.Y.Y.); 2 Mechanical Engineering Department, King Faisal University, P.O. Box 380, Al-Ahsa 31982, Saudi Arabia; maismail@kfu.edu.sa

**Keywords:** orange peel, combustion, TGA, kinetics, model-free, model-fitting, thermodynamic parameters, hemicellulose, cellulose, lignin

## Abstract

This study presents a comprehensive kinetic and thermodynamic investigation of dried orange peel (OP) combustion, employing thermogravimetric analysis (TGA) and differential thermogravimetry (DTG) at high heating rates (20–80 K min^−1^). This gap in high heating rate analysis motivates the novelty of present study, by investigating OP combustion at 20, 40, 60, and 80 K min^−1^ using TGA, to closely simulate rapid thermal conditions typical of industrial combustion processes. Thermal decomposition occurred in three distinct stages corresponding sequentially to the dehydration, degradation of hemicellulose, cellulose, and lignin. Activation energy (*E_a_*) was calculated using six model-free methods—Friedman (FR), Flynn–Wall–Ozawa (FWO), Kissinger–Akahira–Sunose (KAS), Starink (STK), Kissinger (K), and Vyazovkin (VY)—yielding values between 64 and 309 kJ mol^−1^. The *E_a_* increased progressively from the initial to final degradation stages, reflecting the thermal stability differences among biomass constituents. Further kinetic analysis using the Coats–Redfern (CR) model-fitting method identified that first-order (F1), second-order (F2), and diffusion-based mechanisms (D1, D2, D3) effectively describe OP combustion. Calculated thermodynamic parameters—including enthalpy (ΔH), Gibbs free energy (ΔG), and entropy (ΔS)—indicated the endothermic and increasingly non-spontaneous nature of the reactions at higher conversions. These findings demonstrate the potential of OP, an abundant agricultural waste product, as a viable bioenergy resource, contributing valuable insights into sustainable combustion processes.

## 1. Introduction

The escalating global demand for sustainable energy has intensified research into renewable resources, with biomass emerging as a promising carbon-neutral alternative to fossil fuels (Saidur et al. (2013) [1]). Biomass, primarily composed of lignocellulosic materials—cellulose, hemicellulose, and lignin—serves as a versatile feedstock for bioenergy production (Chilla and Suranani (2023) [2]). Among various biomass sources, agricultural waste, such as OP, is particularly attractive due to its abundance and lack of competition with food supply chains, making it a viable candidate for bioenergy applications (Chilla and Suranani (2023) [2]). However, OP utilization faces challenges, including high moisture content, volatile components, and a fibrous structure, all of which affect combustion efficiency (Bridgeman (2008) [3]; Pimchuai (2010) [4]). Most previous studies, however, used relatively low heating rates (≤20 K min^−1^), limiting their direct applicability to rapid industrial-scale biomass combustion systems. To overcome these limitations, thermochemical processes such as combustion, pyrolysis, and gasification are employed, with thermogravimetric analysis (TGA) serving as a key technique for investigating thermal decomposition behavior and reaction kinetics under different atmospheres: nitrogen for pyrolysis (Chilla and Suranani (2023) [2], Lopez-Velazquez et al. (2013) [5]; Anca-Couce et al. (2014) [6]), Santos et al. (2015) [7], Indulekha et al. (2017) [8], Zhu et al. (20211) [9]. Açıkalın (2022) [10], Hosseinzaei et al. (2022) [11], Selvarajoo et al. (2022) [12], Tariq et al. (2022) [13], Kim et al. (2024) [14]), carbon dioxide for gasification (Kwon et al. (2019) [15]; Kim et al. (2024) [14]), and air for combustion (Zapata et al. (2009) [16], Santos (2015) et al. [7], Tariq et al. (2022) [13], Yaradoddi (et al.) (2022) [17], Kim et al. (2024) [14], Kariim et al. (2024) [18]).

Despite extensive studies on OP pyrolysis, its combustion behavior, particularly at high heating rates, remains understudied. Previous studies have provided foundational insights into OP combustion kinetics using TGA. For example, Zapata et al. (2009) [16] employed TG-DSC and TG-FTIR at a single heating rate of 10 K min^−1^, identifying multiple decomposition stages related to hemicellulose, cellulose, and lignin degradation, with activation energies (*E_a_*) ranging from 90 to 190 kJ mol^−1^. Similarly, Santos et al. (2015) [7] conducted non-isothermal TGA at 10–20 K min^−1^, observing three reaction steps up to 1073 K, while Tariq et al. (2022) [13] applied three model-free methods (FWO, KAS, STK) and the Coats-Redfern (CR) approach at 5–20 K min^−1^, reporting *E_a_* values of 70–150 kJ mol^−1^ across a broad conversion range. Yaradoddi et al. (2022) [17] and Kim et al. (2024) [14] further investigated OP combustion at 10 K min^−1^, noting four and three reaction stages, respectively (Table 1). However, these studies primarily employed lower heating rates (5–20 K min^−1^) and applied fewer kinetic models, limiting their ability to capture high-temperature combustion dynamics essential for industrial-scale energy recovery.

This gap in high heating rate analysis motivates the present study, which investigates OP combustion at 20, 40, 60, and 80 K min^−1^ using TGA. These conditions simulate rapid thermal processes relevant to practical combustion systems, providing new insights into OP’s behavior under intensified thermal stress. Given OP’s composition—9.21 wt% cellulose, 0.84 wt% lignin, and 10.50 wt% hemicellulose (Divyabharathi and Subramanian (2021) [19]; López et al. (2010) [20])—it holds significant potential as a biofuel feedstock. To comprehensively characterize its combustion, this work employs six model-free kinetic methods—Friedman (FR), Flynn–Wall–Ozawa (FWO), Kissinger–Akahira–Sunose (KAS), Starink (STK), Kissinger (K), and Vyazovkin (VY)—to determine *E_a_* across conversion levels, extending beyond the three-method approach of Tariq et al. (2022) [13]. Additionally, the CR model-fitting method is applied to identify reaction mechanisms, while thermodynamic parameters (ΔH, ΔG, and ΔS) are evaluated to assess energy feasibility and reaction spontaneity. By expanding the kinetic framework and exploring higher heating rates, this study builds upon previous literature and provides a comprehensive dataset for optimizing OP combustion in sustainable energy applications, with potential extension to other agricultural biomass sources.

## 2. Materials and Methods

### 2.1. OP Material and TGA

OP samples were prepared for thermogravimetric analysis (TGA) by drying at 378 K for 24 h to remove residual moisture, followed by crushing and grinding to a uniform particle size. This preparation ensured consistency and minimized heterogeneity effects during thermal analysis. The proximate and ultimate compositions of the OP samples are detailed in Table 2. Combustion characteristics were evaluated using a thermogravimetric analyzer (TGA) (Mettler Toledo, Columbus, OH, USA) at four heating rates: 20, 40, 60, and 80 K min^−1^. These specific heating rates were selected to represent realistic operating conditions relevant to practical combustion systems. Experiments utilized small sample masses (5–10 mg) to reduce thermal gradients, with temperatures ranging from 298 to 973 K in a controlled airflow of 100 mL min^−1^. To ensure data reliability, TGA runs were conducted in triplicate, achieving a standard deviation of ±2% in mass loss measurements. The instrument was calibrated with standard reference materials to maintain temperature accuracy within ±1 K, and airflow variations were limited to ±5 mL min^−1^ to minimize experimental error. These measures enhanced the reproducibility and precision of the combustion profiles obtained.

### 2.2. Kinetics Equations

The general reaction rate of combustion (*dα*/*dt*) is expressed as a product of the reaction rate constant *k*(*T*) and the reaction model *f*(*α*) according to the following kinetic equation form (Alhulaybi and Dubdub (2024) [21]):

(1) $$\frac{d\alpha }{dt\;}=\beta \frac{d\alpha }{dT}=k\left(T\right)f(\alpha )=A_{0}\;exp(E_{a}/RT)\;f(\alpha )$$

where the conversion rate *α*, is defined as:

(2) $$\alpha =\frac{\left(m_{0}-m_{t}\right)}{\left(m_{0}-m_{f}\right)}$$

The values *m*_0_, *m_t_* and *m_f_* correspond to the initial, intermediate, and final sample masses, respectively. The conversion rate (*α*) expresses the reaction progress, while *k*(*T*) is the reaction rate constant as a function of temperature. This relationship follows the Arrhenius equation, where *E_a_* represents the activation energy (kJ mol^−1^), *A*_0_ is the pre-exponential factor (min^−1^), and *R* denotes the universal gas constant.

(3) $$g(\alpha )\equiv \int_{0}^{\propto }d\alpha /f(\alpha )=(A/\beta )\cdot \int_{0}^{T}exp(-E_{a}/RT)dT$$

where *g*(*α*) represents the reaction model, and the temperature integral in the equation cannot be solved analytically. Consequently, researchers have developed several approximation techniques with varying levels of precision. These techniques can be categorized into differential methods, such as the FR method, and integral methods, including FWO, KAS, STK, VY, and K. These methodologies are commonly referred to as model-free approaches, as they estimate kinetic parameters without assuming a predetermined reaction mechanism. To determine the most appropriate solid-state reaction mechanism, the CR method, classified as a “model-fitting method”, is also applied. Table 3 summarizes the five model-free methods along with the model-fitting method. Additionally, Table 4 lists 15 solid-state thermal reaction mechanisms (*f*(*α*) and *g*(*α*)) utilized in the CR method for evaluating the pre-exponential factor (*A*_0_) in the FR, FWO, KAS, STK, and CR methods.

### 2.3. Thermodynamic Parameters of OP Combustion

The thermodynamic parameters, including ΔH, ΔG and ΔS for OP combustion were calculated using the following equation (Alhulaybi and Dubdub (2024) [21]):

(11) $$H=E_{a}-R\;T_{p}\;$$

(12) $$∆G=E_{a}+R\;T_{p}\ln\left(\frac{k_{B}\;T_{p}}{h\;A_{0}}\right)$$

(13) $$∆S=\frac{∆H-∆G}{T_{p}}$$

where $T_{p}$ represents the maximum temperature (K), $k_{B}$ is the Boltzmann constant (1.381 × 10^−23^ J·K^−1^), h is the Planck constant (6.626 × 10^−34^ J·s), and $A_{0}$ is the pre-exponential factor, (min^−1^). Understanding these parameters is essential for evaluating the feasibility and efficiency of OP combustion (Dhyani et al. (2017) [22]).

## 3. Results and Discussion

### 3.1. TG & DTG Analysis of OP Combustion

**Figure 1:**
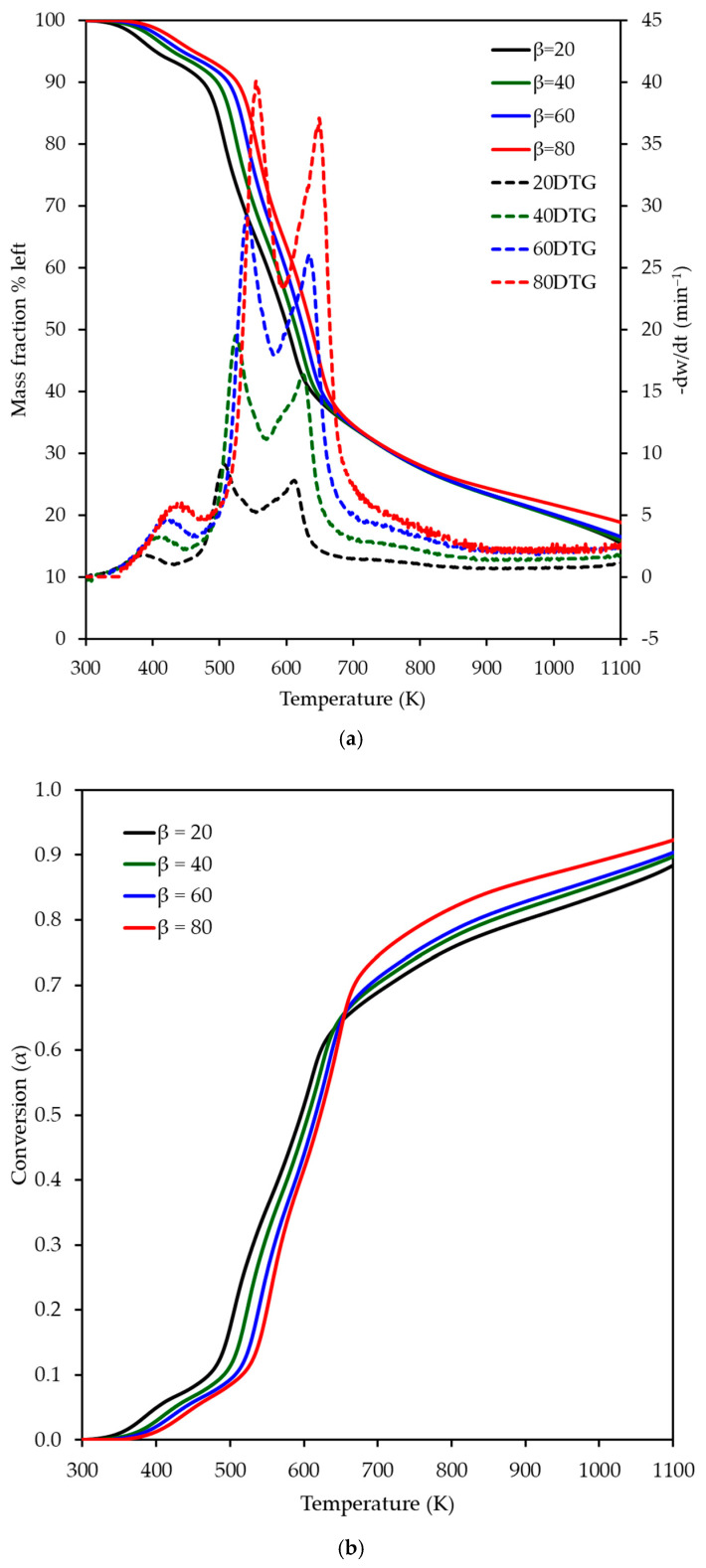
(**a**) Thermogravimetric (TG) and (**b**) derivative thermogravimetric (DTG) curves, showing mass loss and conversion (*α*) profiles for orange peel combustion at heating rates of 20, 40, 60, and 80 K min^−1^.

Thermogravimetric (TG) and derivative thermogravimetric (DTG) analyses of OP combustion at heating rates of 20, 40, 60, and 80 K min^−1^ are presented in Figure 1a,b, illustrating mass loss and conversion profiles as functions of temperature. As the heating rate increased, a shift toward higher temperatures in the TG and DTG curves was observed, reflecting delayed decomposition due to reduced heat transfer efficiency at higher rates. This shift was pronounced up to approximately 65% conversion, beyond which residual char oxidation dominated. Three distinct decomposition stages were identified between 300 and 700 K, corresponding to the dehydration and the sequential degradation of hemicellulose, cellulose, and lignin—key lignocellulosic components of OP. The first stage (362–480 K) involved dehydration with an 8% mass loss, followed by a second stage (436–595 K) attributed to combined hemicellulose and cellulose degradation (25% mass loss), and a third stage (554–732 K) linked to cellulose and lignin breakdown (29% mass loss). These stages align with literature data (Table 1), though the higher heating rates explored here reveal intensified thermal behavior not fully captured in prior studies at lower rates (e.g., 5–20 K min^−1^), highlighting the importance of studying combustion under conditions that mimic industrial thermal processing environments. Kinetic analysis was thus limited to 60% conversion to focus on these primary decomposition phases. Table 5 details the characteristic temperature ranges and mass losses, reinforcing the consistency of these findings with established OP combustion profiles while extending insights into high-temperature dynamics.

### 3.2. Model-Free Methods

The isoconversional model-free approach is frequently employed to estimate kinetic parameters, including activation energy (*E_a_*) and the pre-exponential factor (*A*_0_), from non-isothermal thermogravimetric data. In this work, six well-established model-free techniques were used: FR, FWO, KAS, STK, K, and VY. These methods require data from at least three heating rates and are generally preferred over model-fitting techniques due to their independence from predefined reaction mechanisms. These approaches necessitate data from multiple heating rates and are favored over model-fitting methods, as they eliminate the need to assume a fixed reaction model. The reliability of these methods has been validated by the Kinetics Committee of the International Confederation for Thermal Analysis and Calorimetry (ICTAC) (Ou et al. (2016) [23]).

**Figure 2:**
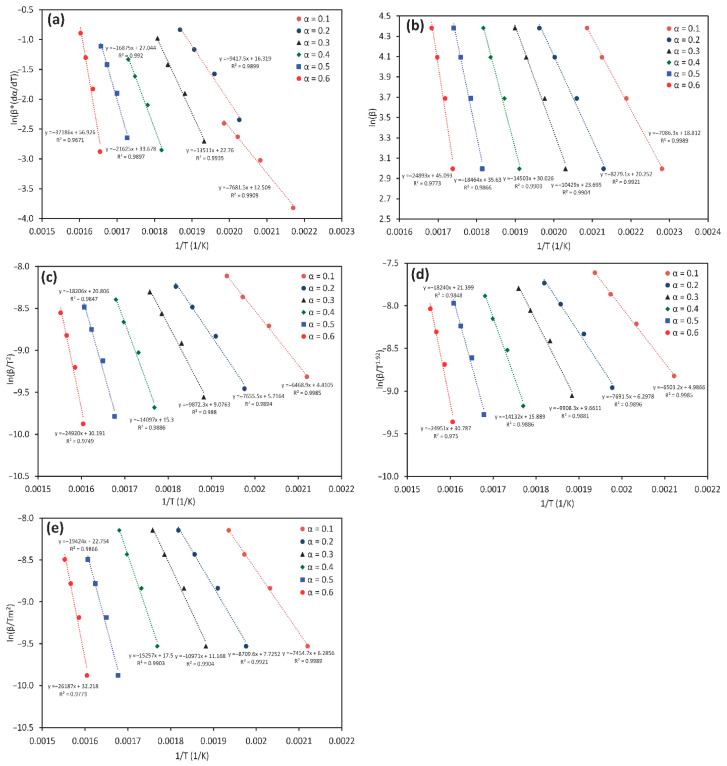
Regression analysis of OP combustion using five model-free methods: (**a**)-FR, (**b**)-FWO, (**c**)-KAS, (**d**)-STK, and (**e**)-K.

**Figure 3:**
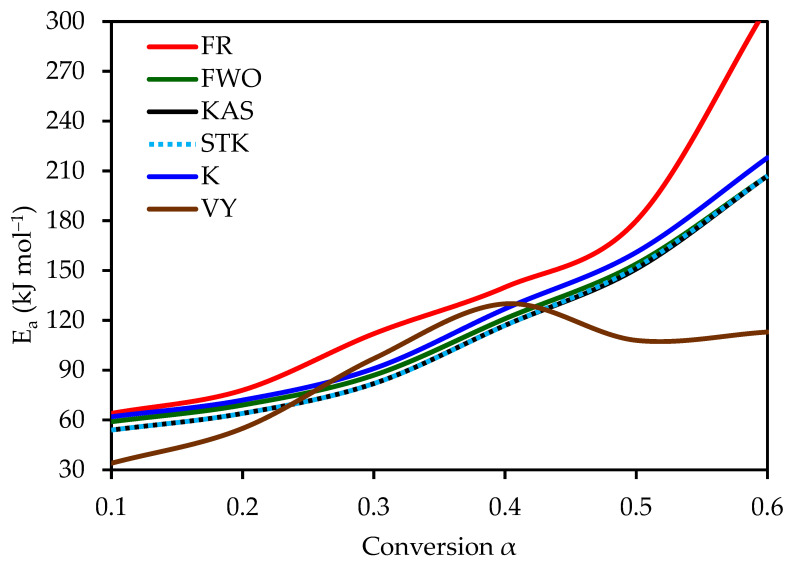
Activation energy (*E_a_*) values of OP combustion determined using six different model-free methods.

The key difference among these model-free methods lies in their underlying assumptions and mathematical formulation (i.e., whether they employ differential or integral approaches). Equations (4)–(8) were used for kinetic calculations, corresponding to the respective linear plots listed in Table 3. For instance, the FWO method involves plotting ln(β) against 1/T. The activation energy (*E_a_*) values were determined over a conversion range of 0.1–0.6, as illustrated in Figure 2. The results in Figure 2 reveal that for each method, the best-fit regression lines shift leftward with increasing conversion from 0.1 to 0.6, indicating a gradual increase in activation energy. This progressive increase in *E_a_* corresponds to the increasing complexity and thermal stability from hemicellulose through cellulose to lignin decomposition. Figure 3 further consolidates these findings, showing that all methods (except FR and VY) exhibit similar trends, thereby confirming the consistency of the calculated activation energy values. A general trend is observed, where the activation energy increases progressively from an average of 55 kJ mol^−1^ at low conversions (*α* = 0.1) to 210 kJ mol^−1^ at higher conversions (*α* = 0.6) (Table 6). This variation is attributed to the transition from hemicellulose degradation to cellulose and lignin decomposition.

Hemicellulose degradation at low conversion levels (*α* = 0.0–0.1) is associated with the lowest activation energy. As the process continues, cellulose degradation occurs within the conversion range of *α* = 0.1–0.4, showing moderate *E_a_* values. Lignin degradation, which takes place at higher conversions (*α* = 0.5–0.6), corresponds to the highest activation energies, reflecting its complex chemical structure. The observed increase in activation energy with conversion indicates that the reaction kinetics are influenced by the physicochemical properties of the biomass components. The decomposition of hemicellulose and cellulose occurs at lower temperatures with lower activation energy, whereas lignin, due to its highly cross-linked aromatic structure, requires higher energy for thermal degradation.

Among various biomass feedstocks, OP has been identified as a highly reactive material, exhibiting one of the lowest ignition temperatures (441–463 K). Comparisons with previous studies indicate that Tariq et al. (2022) [13] observed three DTG peaks in OP combustion, whereas Zapata et al. (2009) reported five peaks, suggesting variability in decomposition pathways under different conditions. They reported that the first one reaction below 373 K is representing the release of water molecules and the last one for char or tar residues degradation, and between them there are for biomass decomposition essentially for hemicellulose, cellulose and lignin. The main differences in the number of peak reactions between these papers may be attributed to: different type of sample and heating rate. The increase in activation energy with conversion is primarily attributed to volatile release and char oxidation. At high conversion levels, *E_a_* increases significantly due to thermal cracking of residual char, which requires higher activation energy for complete oxidation. To advance the kinetic characterization of orange peel (OP) combustion beyond prior work, such as Tariq et al. (2022) [13], this study employs six model-free methods (FR, FWO, KAS, STK, K, VY) compared to their three (FWO, KAS, STK), ensuring a more robust validation of activation energy (*E_a_*) trends across conversions. Additionally, our analysis at elevated heating rates (20–80 K min^−1^ vs. 5–20 K min^−1^ in prior studies) simulates the dynamics of industrial combustion systems, yielding a broader *E_a_* range (64–309 kJ mol^−1^ vs. 70–150 kJ mol^−1^).

Figure 3 and Table 6 highlight that the FR method produces the highest activation energy range (64–309 kJ mol^−1^, average = 147 kJ mol^−1^). The Friedman (FR) method resulted in higher activation energy values (up to 309 kJ mol^−1^) compared to other model-free methods (FWO, KAS, STK, K, VY), which ranged from 55–210 kJ mol^−1^. This discrepancy arises from FR’s differential approach, which uses the reaction rate (*dα*/*dt*) directly, making it sensitive to experimental noise, especially at higher conversions (*α* = 0.6). In contrast, integral methods approximate the temperature integral, providing more stable *E_a_* estimates. The FWO, KAS, STK, and K methods show consistent values within the range of 57–210 kJ mol^−1^, with an average of 116 kJ mol^−1^. The VY method exhibits a narrower range of 55–210 kJ mol^−1^, with an average activation energy of 117 kJ mol^−1^. At high conversion levels, the increased activation energy is indicative of stronger chemical bonding in the residual char, necessitating higher thermal energy for oxidation. These results align closely with previously reported values by Tariq et al. (2022) [13] and Zapata et al. (2009) [16].

From the analysis of the values of *E_a_* against the extent of reaction, Zapata et al. (2009) [16] revealed that there is energetic behavior of different thermal cases during the OP combustion. They found the value of *E_a_* of hemicellulose degradation (90 and 100 kJ mol^−1^), cellulose degradation (120 and 190 kJ mol^−1^), and lignin degradation (115 to 140 kJ mol^−1^) using model-free isoconversional method.

Table 7 presents a comparison of *E_a_* values obtained from two independent studies using model-free methods (K, FWO, KAS, STK). Santos et al. (2015) [7] reported activation energies between 113 and 179 kJ mol^−1^ across three reaction zones. Tariq et al. (2022) [13] documented *E_a_* values between 70 and 140 kJ mol^−1^ over a wider conversion range (*α* = 0.1–0.9). In both studies, the coefficient of determination (*R*^2^) remained above 0.9, confirming the goodness of fit for all kinetic models. The observed differences in *E_a_* values between studies may be attributed to variations in OP composition, experimental conditions, and heating rates. Differences in particle size, moisture content, and oxidative environments can also influence the activation energy values and combustion behavior of OP. The agreement between the findings of this study and previous research demonstrates the robustness of the applied model-free methods in evaluating the combustion kinetics of OP.

The present findings align well with earlier studies on orange peel (OP) combustion but extend the kinetic analysis by covering higher heating rates (20–80 K min^−1^) and employing additional kinetic models. Tariq et al. (2022) [13] reported activation energy (*E_a_*) values between 70 and 150 kJ mol^−1^ at lower heating rates (5–20 K min^−1^) using fewer mod-el-free methods, whereas the current study found broader *E_a_* ranges (64–309 kJ mol^−1^) due to the more intense thermal conditions. Similarly, Zapata et al. (2009) [16] identified multi-ple decomposition stages and activation energies ranging from 90 to 190 kJ mol^−1^ at 10 K min^−1^, consistent with this work’s observations but limited by their single heating rate analysis. This study thus provides a more comprehensive kinetic characterization, im-proving the applicability of results to industrial-scale combustion processes. Uncertainties were quantified using the ±2% mass loss standard deviation from triplicate TGA runs. The activation energy (*E_a_*) has an estimated uncertainty of ±3.5%, with 95% confidence intervals of ±2 kJ mol^−1^ at *α* = 0.1 to ±11 kJ mol^−1^ at *α* = 0.6 (Table 6). Thermodynamic parameters show uncertainties of ±4% for ΔH, ±5% for ΔG, and ±6% for ΔS.

### 3.3. CR Model-Fitting Method

The CR method, a widely recognized model-fitting approach, was employed to determine kinetic parameters, reaction models, and the combustion mechanism of OP. As a model-fitting approach, it requires only a single heating rate experiment to estimate kinetic parameters (Coats and Redfern, 1965 [24]). The CR method (Equation (10)) was applied to determine the activation energy (*E_a_*), pre-exponential factor (*LnA*_0_), and coefficient of determination (*R*^2^) using linear regression analysis at four different heating rates (20, 40, 60, and 80 K min^−1^). The pre-exponential factor (*A*_0_) represents the frequency of reactant molecules engaging in the reaction, serving as an essential parameter in kinetic modelling (Tariq et al., 2022 [13]).

The calculated kinetic parameters for the three reaction zones are summarized in Table 8, which presents the application of the CR method to 15 different reaction models (*g*(*α*)), ranging from F1 to P4. The obtained *R*^2^ values exceed 0.96, confirming the reliability of the model-fitting approach (Galwey (2003) [25]). The CR method was utilized to validate the reaction mechanisms by comparing the activation energy values (*E_a_*) with those obtained from six model-free methods across 15 solid-state reaction mechanisms (F1–P4). The best-fitting mechanisms for OP combustion were identified as first-order (F1) and second-order (F2) reaction order models, as well as one-dimensional, two-dimensional, and three-dimensional diffusion models (D1, D2, and D3) (Tariq et al. (2022) [13]). The three-dimensional diffusion model (D3) consistently provided the highest *R*^2^ values (>0.9968) across all heating rates, clearly indicating that diffusion-controlled processes govern OP combustion.

The values of *E_a_*, *LnA*_0_, and *R*^2^ for these best-fitting mechanisms (D1, D2, and D3) have been highlighted in bold in Table 8. Among these, the three-dimensional diffusion model (D3) was selected for further calculations, including *f*(*α*) and *g*(*α*), as well as the thermodynamic parameters (ΔH, ΔG, and ΔS), using the model-free method (Table 9). The selection of the D3 mechanism suggests that OP combustion is governed by a diffusion-controlled process, where the reaction rate is influenced by the transport of reactants and products through the solid matrix. This finding aligns with previously reported combustion studies on lignocellulosic biomass (Tariq et al., 2022 [13]), further supporting the applicability of the CR method in kinetic modelling of OP combustion.

### 3.4. Thermodynamic Parameters

Thermodynamic parameters—enthalpy (ΔH), entropy (ΔS), and Gibbs free energy (ΔG)—were calculated alongside the pre-exponential factor (*A*_0_) to assess the energy demands of OP combustion, offering insights into reaction mechanisms and energy transfer (Yao et al. (2024) [26]). Each parameter plays a distinct role: ΔH indicates the reaction’s endothermic or exothermic nature, ΔS shows system disorder, and ΔG determines spontaneity. Values were derived using five model-free methods (FR, FWO, KAS, STK, K), with *f*(*α*) and *g*(*α*) based on the three-dimensional diffusion model (D3), as presented in Table 9. Analysis reveals positive ΔH and ΔG across all conversion levels (*α* = 0.1–0.6), while ΔS varies between positive and negative, depending on the stage. Positive ΔG values, especially beyond *α* = 0.3, indicate significant thermodynamic barriers to spontaneous reaction, confirming that external heat input is required for OP combustion.

Table 9 shows ΔH ranging from 50.63 to 303.80 kJ mol^−1^, confirming OP combustion as endothermic, requiring external heat input (Tong et al. (2020) [27]; Huang et al. (2016) [28]). Both ΔH and ΔG increase with conversion, from 50.63–60.63 kJ mol^−1^ and 83.94–143.14 kJ mol^−1^ at *α* = 0.1 to 201.80–303.80 kJ mol^−1^ and 154.64–198.62 kJ mol^−1^ at *α* = 0.6, respectively, reflecting escalating energy needs as decomposition progresses from hemicellulose to lignin. The small *E_a_*–ΔH difference (e.g., ~5–6 kJ mol^−1^, comparing Table 6 and Table 9) suggests a low energy barrier to product formation, facilitating reaction progression (Liu et al., 2022 [29]). However, consistently positive ΔG values, exceeding 130 kJ mol^−1^ beyond *α* = 0.3, indicate non-spontaneity, with a substantial barrier to unassisted reaction (Kalidasan et al. (2023) [30]). Entropy (ΔS) ranges from –0.22842 to 0.20451 kJ mol^−1^ K^−1^, reflecting changes in disorder. Negative ΔS at lower conversions (e.g., –0.2021 to –0.01509 kJ mol^−1^ K^−1^ at *α* = 0.1–0.3) suggests a structured transition state during volatile release, while positive values at *α* = 0.6 (e.g., 0.05359–0.20451 kJ mol^−1^ K^−1^) indicate increased disorder during char oxidation (Ali et al. (2023) [31]). The FWO method yielded slightly lower ΔG (e.g., 83.94 kJ mol^−1^ at *α* = 0.1 vs. 132.48–143.14 kJ mol^−1^ for others), but all methods confirm positive ΔH, reinforcing the thermally driven nature of OP combustion. These results highlight the interplay between kinetic and thermodynamic factors, with endothermic decomposition preceding exothermic char burning, influenced by OP’s high ash content (5.5 wt%, Table 2).

**Table 9:** Pre-exponential factor and thermodynamic parameters (ΔH, ΔG, kJ mol^−1^; ΔS, kJ mol^−1^ K^−1^) for OP combustion, with uncertainties from ±2% mass loss standard deviation.

*α*	FR					FWO				
	*R* ^2^	*A*_0_ (min^−1^)	ΔH (kJ mol^−1^)	ΔG (kJ mol^−1^)	ΔS (kJ mol^−1^)	*R* ^2^	*A*_0_ (min^−1^)	ΔH (kJ mol^−1^)	ΔG (kJ/mol^−1^)	ΔS (kJ/mol^−1^)
0.1	0.9909	6.68 × 10³	60.63	134.57	−0.18255	0.9989	5.13 × 10^9^	55.63	83.94	−0.06989
0.2	0.9899	6.78 × 10^5^	73.64	150.44	−0.1463	0.9921	7.99 × 10^10^	64.64	90.48	−0.04922
0.3	0.9935	7.27 × 10^8^	107.64	153.99	−0.08829	0.9904	4.85 × 10^12^	82.64	90.56	−0.01509
0.4	0.9920	8.16 × 10^10^	135.64	161.38	−0.04904	0.9903	3.82 × 10^15^	116.64	95.45	0.040362
0.5	0.9897	9.23 × 10^13^	174.80	169.82	0.007967	0.9866	1.41 × 10^18^	148.80	93.75	0.088086
0.6	0.9671	1.71 × 10^24^	303.80	175.98	0.204512	0.9773	2.20 × 10^22^	201.80	96.58	0.168352
***α***	**KAS**					**STK**				
	***R* ^2^**	***A*_0_** **(min^−1^)**	**ΔH** **(kJ mol^−1^)**	**ΔG** **(kJ mol^−1^)**	**ΔS** **(kJ mol^−1^)**	***R* ^2^**	***A*_0_** **(min^−1^)**	**ΔH** **(kJ mol^−1^)**	**ΔG** **(kJ mol^−1^)**	**ΔS** **kJ mol^−1^**
0.1	0.9985	6.37 × 10^2^	50.63	132.48	−0.2021	0.9985	2.69 × 10^1^	50.63	143.14	−0.22842
0.2	0.9894	1.20 × 10^4^	59.64	154.05	−0.17983	0.9896	3.63 × 10^2^	59.64	169.32	−0.20893
0.3	0.988	1.08 × 10^6^	77.64	152.39	−0.1424	0.9881	2.00 × 10^4^	77.64	169.82	−0.17559
0.4	0.9886	1.52 × 10^9^	112.64	155.76	−0.08214	0.9886	1.39 × 10^7^	112.64	176.27	−0.12122
0.5	0.9847	8.40 × 10^11^	145.80	165.25	−0.03111	0.9848	4.58 × 10^9^	146.80	193.33	−0.07444
0.6	0.9749	2.23 × 10^16^	201.80	168.31	0.05359	0.975	6.53 × 10^13^	201.80	198.62	0.00509
***α***	**K**									
	***R* ^2^**	***A*_0_** **(min^−1^)**	**ΔH** **(kJ mol^−1^)**	**ΔG** **(kJ mol^−1^)**	**ΔS** **(kJ mol^−1^)**					
0.1	0.9989	4.00 × 10^6^	58.63	111.03	−0.12938					
0.2	0.9921	1.96 × 10^7^	67.64	129.76	−0.11832					
0.3	0.9904	7.75 × 10^8^	86.64	132.71	−0.08775					
0.4	0.9903	6.08 × 10^11^	122.64	139.61	−0.03234					
0.5	0.9866	1.48 × 10^14^	155.80	148.39	0.011866					
0.6	0.9773	2.57 × 10^18^	212.8038	154.6353	0.09307					

Uncertainties: ±4% for ΔH, ±5% for ΔG, ±6% for ΔS.

## 4. Conclusions

This study investigated the combustion kinetics of OP biomass at elevated heating rates (20–80 K min^−1^), representative of industrial thermal processes. Three distinct decomposition stages were identified: hemicellulose degradation (362–480 K; ~8% mass loss), cellulose degradation (436–595 K; ~25% mass loss), and lignin degradation (572–732 K; ~29% mass loss). Kinetic analysis using six model-free methods revealed activation energy values ranging from 64 to 309 kJ mol^−1^, increasing progressively from hemicellulose to lignin decomposition. Further validation using the Coats–Redfern (CR) method indicated that first-order (F1), second-order (F2), and diffusion-controlled mechanisms (D1, D2, D3) accurately describe the combustion process, with the three-dimensional diffusion model (D3) providing the best fit. These kinetic and thermodynamic findings enhance the understanding of OP combustion, underscoring its viability as a sustainable biofuel resource and contributing valuable insights for optimizing biomass combustion in energy production applications. Future studies should focus on scaling these findings for industrial biofuel reactors, considering ash management and emission control technologies to optimize environmental benefits.

## Figures and Tables

**Table 1:** Literature survey of characteristic temperatures and weight losses (%) for OP combustion.

Reference	Heating Rate K min^−1^	1st Reaction			2nd Reaction			3rd Reaction			4th Reaction		
		T Range, T Peak (K)	Weight Loss %	Process	T Range, T Peak (K)	Weight Loss %	Process	T Range, T Peak (K)	Weight Loss %	Process	T Range, T Peak (K)	Weight Loss %	Process
Santos (2015) et al. [7]	10	298–383, 323	10	dehydration	383–498, 473	20	Hemicellulose degradation	498–648, 573	40	Cellulose degradation	648–773, 723	20	Lignin degradation
	15	298–398, 343	10	Cellulose degradation	398–513, 486	20	Hemicellulose degradation	513–663, 593	40	Cellulose degradation	663–778, 733	20	Lignin degradation
	20	298–403, 348	10	Cellulose degradation	403–523, 498	20	Hemicellulose degradation	523–673, 603	40	Cellulose degradation	673–793, 748	20	Lignin degradation
Tariq et al. (2022) [13]	5	298–383, 323	10	Cellulose degradation	383–513, 465	30	Hemicellulose degradation	513–623, 553	30	Cellulose degradation	623–733, 688	20	Lignin degradation
	10	298–403, 333	10	Cellulose degradation	403–523, 477	30	Hemicellulose degradation	523–633, 570	30	Cellulose degradation	633–773, 713	20	Lignin degradation
	15	298–410, 343	10	Cellulose degradation	410–543, 482	30	Hemicellulose degradation	543–643, 573	30	Cellulose degradation	643–783, 705	20	Lignin degradation
	20	298–423, 353	10	Cellulose degradation	423–553, 491		Hemicellulose degradation	553–653, 585	30	Cellulose degradation	653–793, 723	20	Lignin degradation
Yaradoddi (et al.) (2022) [17]	10	315–446, 364	12.5	Cellulose degradation	447–501, 473	59.4	Hemicellulose & Cellulose degradation	501–607, 553	10.6	NA *	573–673, 623	4.5	Lignin degradation
Kim et al. (2024) [14]	10	298–373, 343	10	NA *	373–543, 498	20	NA *	543–673, 580	40	NA *	673,	25	NA *

*: Not available.

**Table 2:** OP composition: Proximate and ultimate analyses.

Proximate Analysis (wt.%)		Ultimate Analysis (wt.%)	
Moisture content	5.03	C	36.05
Volatile matter	69.5	H	3.601
Ash	5.5	N	13.27
Fixed carbon ^a^	19.9	S	3.901
		O ^b^	43.176

^a^ Fixed carbon is calculated by difference: 100 − (Moisture + Volatile Matter + Ash) %. ^b^ Oxygen content is determined by difference: 100 − (C + H + N + S) %.

**Table 3:** Summary of model-free and model-fitting methods used for the kinetic analysis of OP combustion, including their corresponding equations and regression plots (Alhulaybi and Dubdub, 2024 [21]).

Model-Free Methods			
Method	Formula		Plot
FR	$\ln\left(\beta \frac{d\alpha }{dT}\right)=\ln\left[A_{0}f(\alpha \right)]-\frac{E_{a}}{RT}$	(4)	$\ln\left(\beta \frac{d\alpha }{dT}\right)\;vs.\;\;\frac{1}{T}$
FWO	$\ln\left(\beta \right)=\ln\frac{A_{0}E_{a}}{Rg(\alpha )}-5.331-1.052\frac{E_{a}}{RT}$	(5)	$\ln\left(\beta \right)\;vs.\;\;\frac{1}{T}$
KAS	$\ln\left(\frac{\beta }{T^{2}}\right)=\ln\frac{A_{0}R}{E_{a}\;g(\alpha )}-\frac{E_{a}}{RT}$	(6)	$\ln\left(\frac{\beta }{T^{2}}\right)\;vs.\;\;\frac{1}{T}$
STK	$\ln\frac{\beta }{T^{1.92}}=\ln\left(\frac{A_{0}E_{a}}{Rg(\alpha )}\right)-1.0008\frac{E_{a}}{RT}$	(7)	$ln\frac{\beta }{T^{1.92}}\;vs.\;\;\frac{1}{T}$
K	$\ln(\frac{\beta }{T_{m}^{2}})=\ln\left(\frac{A_{0}R}{E_{a}}\right)-\frac{E_{a}}{RT}$	(8)	$\ln(\frac{\beta }{T_{m}^{2}})\;vs.\;\;\frac{1}{T}$
VY	$\Phi \left(E_{\alpha }\right)=\sum_{i=1}^{n}\sum_{j\neq i}^{n}\frac{J[E_{\alpha },\;T_{i}\left(t_{\alpha }\right)]}{J[E_{\alpha },\;T_{j}\left(t_{\alpha }\right)]}=0$	(9)	minimizing the function $\Phi \left(E_{\alpha }\right)$
Model-fitting methods			
Method	Formula		Plot
CR	$ln\left[\frac{g(\alpha )}{T^{2}}\right]=ln\left[\frac{A_{0}R}{\beta E_{a}}\right]-\frac{E}{RT}$	(10)	$ln\left[\frac{g(\alpha )}{T^{2}}\right]\;vs.\;\frac{1}{T}$

**Table 4:** Sixteen solid-state reaction mechanisms (Alhulaybi and Dubdub, 2024 [21]).

Reaction Mechanism	Code	*f*(*α*)	*g*(*α*)
Reaction order models–First order	F1	1 − *α*	−ln⁡(1−α)
Reaction order models–Second order	F2	${\left(1-\alpha \right)}^{2}$	${\left(1-\alpha \right)}^{-1}-1$
Reaction order models–Third order	F3	${\left(1-\alpha \right)}^{3}$	${\left[(1-\alpha \right)}^{-1}-1]/2$
Diffusion model–One-dimensional	D1	$1/2\alpha^{-1}$	$\alpha^{2}$
Diffusion model–Two-dimensional	D2	${\left[-\ln(1-\alpha )\right]}^{-1}$	$\left(1-\alpha \right)\ln\left(1-\alpha \right)+\alpha$
Diffusion model–Three-dimensional	D3	$3/2{\left[1-{\left(1-\alpha \right)}^{1/3}\right]}^{-1}$	${\left[1-{\left(1-\alpha \right)}^{1/3}\right]}^{2}$
Diffusion model–Four-dimensional	D4	1.5 × [(1 − *α*)^1/3^ – 1]	1 − (2/3) × *α* − (1 − *α*)^2/3^
Nucleation models–Two-dimensional	A2	$2(1-\alpha ){\left[-\ln\left(1-\alpha \right)\right]}^{1/2}$	${\left[-\ln\left(1-\alpha \right)\right]}^{1/2}$
Nucleation models–Three-dimensional	A3	$3(1-\alpha ){\left[-\ln\left(1-\alpha \right)\right]}^{1/3}$	${\left[-\ln\left(1-\alpha \right)\right]}^{1/3}$
Nucleation models–Four-dimensional	A4	$4(1-\alpha ){\left[-\ln\left(1-\alpha \right)\right]}^{1/4}$	${\left[-\ln\left(1-\alpha \right)\right]}^{1/4}$
Geometrical contraction models–One-dimensional;	R1	1	α
Geometrical contraction models—Sphere	R2	$2{\left(1-\alpha \right)}^{1/2}$	$1-{\left(1-\alpha \right)}^{1/2}$
Geometrical contraction models—Cylinder	R3	$3{\left(1-\alpha \right)}^{1/3}$	$1-{\left(1-\alpha \right)}^{1/3}$
Nucleation models–2-Power law	P2	$2\alpha^{1/2}$	$\alpha^{1/2}$
Nucleation models–3-Power law	P3	$3\alpha^{2/3}$	$\alpha^{1/3}$
Nucleation models–4-Power law	P4	$4\alpha^{3/4}$	$\alpha^{1/4}$

**Table 5:** Characteristic temperatures and weight losses (%) for OP combustion obtained in this study.

Heating Rate (K min^−1^)	1st Reaction			2nd Reaction			3rd Reaction		
	T Range, T Peak (K)	Weight Loss %	Process	T Range, T Peak (K)	Weight Loss %	Process	T Range, T Peak (K)	Weight Loss %	Process
**20**	362–436, 380	8	Dehydration	436–554, 514	25	Hemicellulose & Cellulose degradation	554–666, 616	29	Cellulose & Lignin degradation
**40**	364–454, 416	8	Dehydration	454–572, 526	25	Hemicellulose & Cellulose degradation	572–692, 628	29	Cellulose & Lignin degradation
**60**	378–464, 420	8	Dehydration	464–586, 541	25	Hemicellulose & Cellulose degradation	586–698, 638	29	Cellulose & Lignin degradation
**80**	380–480, 438	8	Dehydration	480–595, 553	25	Hemicellulose & Cellulose degradation	595–732, 650	29	Cellulose & Lignin degradation

**Table 6:** Kinetic parameter values obtained using six model-free methods for OP combustion at different conversion levels.

Conversion	FR		FWO		KAS		STK		K		VY		Average	
	*E* (kJ mol^−1^)	*R* ^2^	*E* (kJ mol^−1^)	*R* ^2^	*E* (kJ mol^−1^)	*R* ^2^	*E* (kJ mol^−1^)	*R* ^2^	*E* (kJ mol^−1^)	*R* ^2^	*E* (kJ mol^−1^)	*R* ^2^	*E* (kJ mol^−1^)	*R* ^2^
0.1	64	0.9909	59	0.9989	54	0.9985	54	0.9985	62	0.9989	34	NA *	55	0.9005
0.2	78	0.9899	69	0.9921	64	0.9894	64	0.9896	72	0.9921	55	NA *	67	0.9408
0.3	112	0.9935	87	0.9904	82	0.988	82	0.9881	91	0.9904	97	NA *	92	0.9659
0.4	140	0.992	121	0.9903	117	0.9886	117	0.9886	127	0.9903	130	NA *	125	0.9879
0.5	180	0.9897	154	0.9866	151	0.9847	152	0.9848	161	0.9866	108	NA *	151	0.9900
0.6	309	0.9671	207	0.9773	207	0.9749	207	0.975	218	0.9773	113	NA *	210	0.9971
Average	147	0.9872	116	0.9893	113	0.9874	113	0.9874	122	0.9893	90	NA *	117	0.9712

*: Not available; Uncertainties: *E_a_* ±3.5% (95% CI: ±2–11 kJ mol^−1^), *ln*(*A*_0_) ± 4%.

**Table 7:** Activation energy values by previous researchers obtained by different model-free methods fr OP combustion.

Reference	Model-Free Method	1st Reaction			2nd Reaction			3rd Reaction		
		*E_a_* (kJ mol^−1^)	*R* ^2^		*E_a_* (kJ mol^−1^)	*R* ^2^		*E_a_* (kJ mol^−1^)	*R* ^2^	
Santos et al. (2015) [7]	K	113	0.9976		121	0.9579		179	0.9709	
Tariq et al. (2022) [13]		*E_a_* (kJ mol^−1^)								
		*α* = 0.1	0.2	0.3	0.4	0.5	0.6	0.7	0.8	0.9
	FWO	70	105	130	150	130	100	80	140	125
	KAS	80	105	120	125	110	100	75	120	110
	STK	75	105	120	125	120	100	75	125	125

**Table 8:** Kinetic parameters obtained by the CR method of OP combustion for four heating rates.

Reaction mechanism 1-step reaction	Code	20			40			60		
		*E_a_* (kJ mol^−1^)	*Ln*(*A*_0_)	*R* ^2^	*E_a_* (kJ mol^−1^)	*Ln*(*A*_0_)	*R* ^2^	*E_a_* (kJ mol^−1^)	*Ln*(*A*_0_)	*R* ^2^
Reaction order models–First order	F1	29	17.34	0.996	33	17.52	0.9972	32	18.71	0.9977
Reaction order models–Second order	F2	30	17.18	0.9962	34	17.37	0.9973	32	18.53	0.9978
Reaction order models–Third order	F3	30	16.98	0.9965	34	17.19	0.9975	33	18.38	0.9979
**Diffusion models–One-dimensional**	**D1**	**63**	**13.06**	**0.9967**	**72**	**15.3**	**0.9976**	**69**	**14.07**	**0.9981**
**Diffusion models–Two-dimensional**	**D2**	**64**	**12.51**	**0.9967**	**72**	**14.72**	**0.9976**	**69**	**13.5**	**0.9981**
**Diffusion models–Three-dimensional**	**D3**	**64**	**12.76**	**0.9968**	**73**	**13.34**	**0.9977**	**70**	**14.14**	**0.9981**
Diffusion models–Four-dimensional	D4	64	12.84	0.9967	73	13.27	0.9976	6	22.16	0.992
Nucleation models–Two-dimensional	A2	11	20.22	0.9931	13	20.8	0.9953	12	21.57	0.996
Nucleation models–Three-dimensional	A3	5	20.71	0.9861	7	21.59	0.9912	6	22.16	0.992
Nucleation models–Four-dimensional	A4	2	20.44	0.9618	3	21.44	0.9794	3	22.11	0.978
Geometrical contraction models–One-dimensional phase boundary	R1	28	17.49	0.9958	33	17.69	0.997	31	18.86	0.9976
Geometrical contraction models–Contracting sphere	R2	29	18.13	0.9959	33	18.3	0.9971	31	19.47	0.9976
Geometrical contraction models–Contracting cylinder	R3	29	18.5	0.9959	33	18.67	0.9971	31	19.84	0.9976
Nucleation models–Power law	P2	11	20.31	0.9926	13	20.89	0.9949	12	21.67	0.9957
Nucleation models–Power law	P3	5	20.78	0.9848	6	21.49	0.9904	6	22.22	0.9913
Nucleation models–Power law	P4	2	20.49	0.9565	3	21.49	0.9771	3	22.15	0.9751
**Reaction mechanism 1-step reaction**	**Code**	**80**								
		***E_a_*** **(kJ mol^−1^)**	***Ln*(*A*_0_)**	***R* ^2^**						
Reaction order models–First order	F1	34	18.61	0.9979						
Reaction order models–Second order	F2	35	18.45	0.998						
Reaction order models–Third order	F3	36	18.3	0.9981						
**Diffusion models–One-dimensional**	**D1**	**71**	**15.36**	**0.9982**						
**Diffusion models–Two-dimensional**	**D2**	**75**	**14.84**	**0.9982**						
**Diffusion models–Three-dimensional**	**D3**	**76**	**13.54**	**0.9983**						
Diffusion models–Four-dimensional	D4	75	13.6	0.9982						
Nucleation models–Two-dimensional	A2	14	21.82	0.9964						
Nucleation models–Three-dimensional	A3	7	22.49	0.9932						
Nucleation models–Four-dimensional	A4	3	23.32	0.9832						
Geometrical contraction models–One-dimensional phase boundary	R1	34	18.79	0.9977						
Geometrical contraction models–Contracting sphere	R2	34	19.39	0.9978						
Geometrical contraction models–Contracting cylinder	R3	34	19.77	0.9978						
Nucleation models–Power law	P2	13	21.83	0.9962						
Nucleation models–Power law	P3	7	22.46	0.9926						
Nucleation models–Power law	P4	3	22.37	0.9812						
**Reaction mechanism 2-step reaction**	**Code**	**20**			**40**			**60**		
		***E_a_*** **(kJ mol^−1^)**	***Ln*(*A*_0_)**	***R* ^2^**	***E_a_*** **(kJ mol^−1^)**	***Ln*(*A*_0_)**	***R* ^2^**	***E_a_*** **(kJ mol^−1^)**	***Ln*(*A*_0_)**	***R* ^2^**
Reaction order models–First order	F1	43	15.35	0.9928	55	13.9	0.9972	58	14.13	0.9971
Reaction order models–Second order	F2	49	13.83	0.9945	61	12.66	0.9964	66	13.82	0.998
Reaction order models–Third order	F3	56	12.19	0.9957	67	14.32	0.9956	75	16.03	0.9987
**Diffusion models–One-dimensional**	**D1**	**82**	**16.01**	**0.9925**	**107**	**22.04**	**0.9981**	**109**	**22.2**	**0.9966**
**Diffusion models–Two-dimensional**	**D2**	**86**	**16.34**	**0.9931**	**111**	**22.28**	**0.9979**	**114**	**22.7**	**0.9969**
**Diffusion models–Three-dimensional**	**D3**	**90**	**15.9**	**0.9936**	**115**	**21.75**	**0.9977**	**119**	**22.45**	**0.9973**
Diffusion models–Four-dimensional	D4	87	15.19	0.9932	112	21.1	0.9979	116	21.62	0.997
Nucleation models–Two-dimensional	A2	17	19.75	0.9885	23	19.57	0.9961	24	19.95	0.9959
Nucleation models–Three-dimensional	A3	9	20.9	0.9794	13	21.17	0.9944	13	21.57	0.9936
Nucleation models–Four-dimensional	A4	4	20.97	0.9543	7	21.65	0.9909	8	22.3	0.9892
Geometrical contraction models–One-dimensional phase boundary	R1	37	16.72	0.9906	49	15.17	0.9978	50	15.78	0.9959
Geometrical contraction models–Contracting sphere	R2	40	16.75	0.9917	52	15.24	0.9975	54	15.67	0.9965
Geometrical contraction models–Contracting cylinder	R3	41	16.92	0.9921	53	15.43	0.9974	55	15.79	0.9968
Nucleation models–Power law	P2	1	17.68	0.9879	20	20.12	0.9969	21	20.71	0.9937
Nucleation models–Power law	P3	7	21.15	0.9666	11	21.47	0.9952	11	22	0.9895
Nucleation models–Power law	P4	3	21.07	0.9025	6	21.84	0.9914	6	22.36	0.9796
**Reaction mechanism 2-step reaction**	**Code**	**80**								
		***E_a_*** **(kJ mol^−1^)**	***Ln*(*A*_0_)**	***R* ^2^**						
Reaction order models–First order	F1	62	13.91	0.9979						
Reaction order models–Second order	F2	70	14.61	0.9973						
Reaction order models–Third order	F3	78	16.64	0.9965						
**Diffusion models–One-dimensional**	**D1**	**118**	**23.98**	**0.9985**						
**Diffusion models–Two-dimensional**	**D2**	**123**	**24.4**	**0.9984**						
**Diffusion models–Three-dimensional**	**D3**	**128**	**24.07**	**0.9983**						
Diffusion models–Four-dimensional	D4	125	23.29	0.9984						
Nucleation models–Two-dimensional	A2	26	20.04	0.9972						
Nucleation models–Three-dimensional	A3	15	21.82	0.9961						
Nucleation models–Four-dimensional	A4	9	22.48	0.9939						
Geometrical contraction models–One-dimensional phase boundary	R1	55	15.47	0.9982						
Geometrical contraction models–Contracting sphere	R2	58	15.39	0.9981						
Geometrical contraction models–Contracting cylinder	R3	59	15.53	0.9981						
Nucleation models–Power law	P2	23	20.76	0.9973						
Nucleation models–Power law	P3	12	22.16	0.9961						
Nucleation models–Power law	P4	7	22.65	0.9932						
**Reaction mechanism 3-step reaction**	**Code**	**20**			**40**			**60**		
		***E_a_*** **(kJ mol^−1^)**	***Ln*(*A*_0_)**	***R* ^2^**	***E_a_*** **(kJ mol^−1^)**	***Ln*(*A*_0_)**	***R* ^2^**	***E_a_*** **(kJ mol^−1^)**	***Ln*(*A*_0_)**	***R* ^2^**
Reaction order models–First order	F1	20	19.86	0.9974	25	19.9	0.9981	28	19.89	0.9983
Reaction order models–Second order	F2	31	17.66	0.9958	40	17.05	0.9969	45	16.65	0.9962
Reaction order models–Third order	F3	45	14.91	0.9945	58	13.44	0.9959	66	13.59	0.994
**Diffusion models–One-dimensional**	**D1**	**32**	**18.93**	**0.9995**	**37**	**18.92**	**0.9997**	**41**	**18.86**	**0.9986**
**Diffusion models–Two-dimensional**	**D2**	**37**	**18.5**	**0.9991**	**44**	**18.25**	**0.9994**	**48**	**18.04**	**0.999**
**Diffusion models–Three-dimensional**	**D3**	**44**	**18.7**	**0.9986**	**52**	**18.1**	**0.999**	**57**	**17.71**	**0.999**
Diffusion models–Four-dimensional	D4	39	19.56	0.999	47	19.22	0.9993	3	22.46	0.9805
Nucleation models–Two-dimensional	A2	5	21.32	0.9914	7	21.94	0.9953	9	22.36	0.9962
Nucleation models–Three-dimensional	A3	NA *	NA *	NA *	2	21.8	0.9597	3	22.46	0.9805
Nucleation models–Four-dimensional	A4	NA *	NA *	NA *	1	21.66	0.9736	NA *	NA *	NA *
Geometrical contraction models–One-dimensional phase boundary	R1	11	21.41	0.9991	13	21.89	0.9995	15	22.16	0.9972
Geometrical contraction models–Contracting sphere	R2	15	21.4	0.9982	19	21.72	0.9988	21	21.85	0.9986
Geometrical contraction models–Contracting cylinder	R3	17	21.57	0.9979	21	21.78	0.9986	24	21.89	0.9986
Nucleation models–Power law	P2	1	20.78	0.9626	2	22.01	0.9945	2	22.3	0.968
Nucleation models–Power law	P3	NA *	NA *	NA *	NA *	NA *	0.9993	NA *	NA *	NA *
Nucleation models–Power law	P4	NA *	NA *	NA *	NA *	NA *	0.9999	NA *	NA *	NA *
**Reaction mechanism 3-step reaction**	**Code**	**80**								
		***E_a_*** **(kJ mol^−1^)**	***Ln*(*A*_0_)**	***R* ^2^**						
Reaction order models–First order	F1	33	19.46	0.9974						
Reaction order models–Second order	F2	54	15.6	0.9954						
Reaction order models–Third order	F3	79	16.4	0.9938						
**Diffusion models–One-dimensional**	**D1**	**46**	**18.39**	**0.9996**						
**Diffusion models–Two-dimensional**	**D2**	**57**	**17.38**	**0.9992**						
**Diffusion models–Three-dimensional**	**D3**	**66**	**16.7**	**0.9986**						
Diffusion models–Four-dimensional	D4	82	14.17	0.9996						
Nucleation models–Two-dimensional	A2	11	22.42	0.9949						
Nucleation models–Three-dimensional	A3	4	22.76	0.9836						
Nucleation models–Four-dimensional	A4	1	22.05	0.5383						
Geometrical contraction models–One-dimensional phase boundary	R1	18	22.21	0.9994						
Geometrical contraction models–Contracting sphere	R2	25	21.68	0.9985						
Geometrical contraction models–Contracting cylinder	R3	28	21.63	0.9981						
Nucleation models–Power law	P2	NA *	NA *	0.9975						
Nucleation models–Power law	P3	NA *	NA *	0.9997						
Nucleation models–Power law	P4	NA *	NA *	0.9931						

*: Not available.

## Data Availability

The original contributions presented in this study are included in the article. Further inquiries can be directed to the corresponding author.
